# Effects of 1‐methylcyclopropene on the quality attributes of harvested Chinese mushroom (*Volvariella volvacea*) fruiting bodies

**DOI:** 10.1002/fsn3.919

**Published:** 2019-01-22

**Authors:** Bingzhi Chen, Guohong Wu, Ling Li, Qi Wei, Qiangui Zhong, Xiaoning Chen, Kaiqian Xiao, Baogui Xie, Yuji Jiang

**Affiliations:** ^1^ College of Food Science Fujian Agriculture and Forestry University Fuzhou China; ^2^ Department of Food and Drag Qingyuan Polytechnic Qingyuan China; ^3^ Mycological Research Center College of life sciences Fujian Agriculture and Forestry University Fuzhou China

**Keywords:** 1‐methylcyclopropene, browning, Chinese mushroom, ethylene receptor inhibitor, mushroom quality

## Abstract

The effects of paper containing different concentrations of 1‐methylcyclopropene (1‐MCP) on the quality of Chinese mushrooms were investigated. Mushrooms were first stored with paper containing 0, 0.25, 0.50, 0.75, 1.00, or 1.25 μl L^−1^ 1‐MCP at 15 ± 1°C for 12 hr and then continued for up to 8 days. 1‐MCP reduced the respiratory peak, weight loss, degree of cap browning, relative leakage rate, and soluble solid content, and delayed the change in the surface hue angle. Mushrooms exposed to paper containing 1‐MCP maintained firmness and sensory attributes. The storage time of Chinese mushrooms treated with 1‐MCP at the best concentration (0.75 μl L^−1^) was 8 days, which was 4 days longer than that of the control. Browning in mushrooms was related to the vitamin C content, total phenol content, and polyphenol oxidase activity. The results would be very useful to the mushroom industry and provide a theoretical basis for the potential researches of the mechanisms maturation and senescence in Chinese mushrooms.

## 1 INTRODUCTION

*Volvariella volvacea* (Bull, ex Fr.) Sing., also known as the Chinese mushroom (CM), straw mushroom, or the king of mushrooms, is an edible fungus that grows and fruits at high temperature (28–35°C). It is considered a delicious and juicy mushroom that is rich in nutrients and has medicinal value (Chang & Yau, 1971; Diamantopoulou, Papanikolaou, Aggelis, & Philippoussis, 2016). The yield of CM ranks sixth among the worldwide mushroom production (Zhang, 2009). During storage of CM, changes in respiration rate are similar to those typical for a climacteric variant, with a respiration peak on day 2 of storage (Xie, Xie, Lin, Yi, & Fu, 2005). CM is typically cultivated in summer, with the optimal growing season spanning from May to September. However, it is the most difficult to preserve among all mushrooms because the pileus opens readily after harvest, which results in loss of nutritional and commodity values. In summer, the mushrooms can be stored for only 1–2 days at room temperature, and storage at <10°C causes fruit body autolysis and browning atrophy leading to unpleasant odors. These challenges shorten mushroom storage life and restrict their distribution.

Currently, CM is routinely preserved under temperature control at 15 ± 1°C, which increases the shelf life to 2–3 days (Wu et al., 2004), or by using chemical preservatives, such as sodium dehydroacetate (Hou et al., 2014). Other methods to increase storage life of mushrooms include ozone (Dong, Chui, Wang, & Zhong, 1998), irradiation (Xie et al., 2005), ultrasound treatment, and control of relative humidity (Li et al., 2017). However, these methods are not used commercially.

1‐Methylcyclopropene (1‐MCP) is a competitive inhibitor that can inhibit ethylene signal transduction pathways (Blankenship & Dole, 2003). It has been found to prolong the shelf life of many fruits, vegetables, and flowers (Blankenship & Dole, 2003; Chen et al., 2015; Li et al., 2016; Storch et al., 2017; Watkins, 2006). Recently, 1‐MCP has also been used on mushrooms. Huang, Liu, He, and Zhang (2010) used 0, 10, and 100 μg L^−1^ 1‐MCP powder to treat *Agaricus bisporus* and found that 10 μg L^−1^ 1‐MCP had positive impacts on changes in peroxidase and superoxide dismutase activities, antioxidants, and other quality parameters. Zhao, Ma, Feng, and Liu (2012) used 0.1, 0.3, and 0.5 μL L^−1^ 1‐MCP powder to treat *Pleurotus eryngii* and found that 0.3 μg L^−1^ 1‐MCP was optimal for slowing down weight loss and for reducing browning and softening. Jamjumroon et al. (2013) treated *V. volvacea* with 250, 500, and 1000 ppb 1‐MCP and found that 250 ppb 1‐MCP at 25°C for 6 hr reduced browning and increased the shelf life of the straw mushrooms. However, 1‐MCP powder is inconvenient for postharvest treatment of mushrooms because it requires accurate quantification, and weighing instruments are generally not available in the postharvest handling environment, especially in mushroom production houses (Chen et al., 2015).

Our study in 2017 demonstrated that paper containing 0.25 μl L^−1^ 1‐MCP is suitable for the preservation of CM after comparing six common preservation methods (Wu & Jiang, 2017). However, only one 1‐MCP concentration was tested, and the optimal concentration was not determined. In addition, few indexes were evaluated. Therefore, in this study, Chinese mushrooms were stored in paper containing different concentrations of 1‐MCP at 15 ± 1°C. The sensory quality, weight, firmness, leakage rate, surface hue angle, browning degree, and respiration rate were measured to determine whether paper containing 1‐MCP can increase the storage life and which concentration has the best preservation effect of CM.

## 2 MATERIALS AND METHODS

### 2.1 Materials and treatments

CM was purchased from Lvbao Agricultural Development Co., Ltd. in Youxi County, Sanming City, Fujian, China. One thousand nonmechanically damaged egg‐stage Chinese mushrooms of uniform size and color were selected. One hundred mushrooms were used to determine the mushroom properties at harvest (day 0), and the remaining mushrooms were randomly divided into six groups of 150 mushrooms each for following treatments in triplicate. Mushrooms were precooled at 15 ± 1°C for 2 hr and then stored in paper containing 0, 0.25, 0.50, 0.75, 1.00, or 1.25 μl L^−1^ 1‐MCP (AnsiP‐S, a new type of 1‐MCP product; Litong Co., Ltd., Shanghai, China) for 12 hr. After treatments, the Chinese mushrooms were placed in 0.02‐mm‐thick polyethylene bags and stored at 15 ± 1°C for 8 days.

### 2.2 Measurement of weight loss and firmness

Five bags (10 mushrooms per bag) from each treatment were chosen to measure the weight loss using the following formula: percentage of weight loss = [(pre‐storage fruit weight – fruit weight after storage)/pre‐storage fruit weight] × 100%.

Ten mushrooms from each treatment were used to measure the firmness according to the method described by Luo, Xie, Xu, and Zhang (2009). TA‐XT2i astral instrument was used to test the firmness of each mushroom on the Tropic of Capricorn 3 position. The firmness was determined using a 15‐mm probe radius by compressing the mushroom sample to 10 mm at a 1 mm s^−1^ speed. Maximum compression readings were averaged.

### 2.3 Measurement of relative leakage of cellular membrane, respiration rate, and surface hue angle

Thirty mushrooms from each treatment were used to determine ion leakage as described by Chen et al. (2014) to express the level of membrane permeability in mushrooms. During storage, every day, three mushrooms were cut to discs of 5 mm in diameter and 2 mm thickness and the three mushrooms discs of approximately 2.0 g were rinsed twice and then soaked in 25 mL of distilled water at 25°C for 2 hr. The conductivity of the leaching liquid (C1) was measured using an electrical conductivity meter (Model 3173; Shanghai Electronics Co., Ltd., Shanghai, China). Then, the samples were boiled for 30 min in 25 mL of distilled water and cooled rapidly to 25°C to assess total electrolytes (C2). Relative leakage (%) was expressed as (C1/C2) × 100%.

The respiration rate was determined in five mushrooms for each treatment as described by Chen et al. (2015). The CO_2_ concentration was directly measured using an infrared gas analyzer (GXH‐3010D; Beijing Computer Technology and Application Institute) at 15 ± 1°C and was expressed in mg kg^−1^ hr^−1^.

The surface hue angle (h°) was determined using an ADCI colorimeter (ADCI‐60‐C; Beijing Chentaike Instrument Technology Co., Ltd., Beijing, China). Five mushrooms were randomly chosen from each treatment and were measured at four equidistant points from the cap of the mushroom. The hue angle (h°) was calculated per the following equation: h° = 180° + arctan (b/a), a * (redness) and b * (yellowness) (Shi, Cui, Yin, Luo, & Zhou, 2014).

### 2.4 Measurement of soluble solid content

Thirty mushrooms were randomly chosen from each treatment, and the soluble solid contents were measured using a refractometer (WYT‐1; Chengdu Taihua Optical Co., Ltd., China).

### 2.5 Measurement of browning degree

The browning degree of five mushrooms per treatment was evaluated as described by Su, Jiang, Li, and Lin (2003). Mushrooms were homogenized at 4°C in distilled water at a l: 10 weight ratio. The filtrate was left at 25°C for 5 min and then diluted 1 time. The browning degree was expressed as the absorbance of the diluted filtrate at 410 nm (A_410_ * 10).

### 2.6 Sensory quality analysis

As described by Wu et al. (2014), a panel of up to 10 people evaluated the sensory quality of mushrooms stored with different concentrations of paper containing 1‐MCP in terms of appearance, color, texture, and flavor. Each characteristic was scored on a scale of 0–10, with 10 representing fresh mushroom and 0 putrefied mushroom.

### 2.7 Measurement of vitamin C (Vc) content, total phenol content, and polyphenol oxidase (PPO) activity

Fifteen mushrooms per treatment were used to evaluate the Vc content, total phenol content, and PPO activity. The mushrooms were divided into three repeat groups (two mushrooms per repeat group). Vc content was measured according to the method of Arakawa, Tsutsumi, Sanceda, Kurata, and Inagaki (1981), with a slight modification. Fresh mushrooms were cut and blended, and 2.0 g was added to 15 ml of precooled 5% trichloroacetic acid (TCA) solution. The mixture was ground on ice and centrifuged at 15,000 × *g* for 20 min at 4 °C. The supernatant was removed, and TCA solution was added to a volume of 25 ml. After mixing, 0.5 ml of this crude Vc extract was added to 1.5 ml of 5% TCA solution and 1 ml of anhydrous ethanol, and Vc was detected by the 4,7‐diphenyl‐1,10‐phenanthroline method.

Total phenol was measured according to the method of Beta, Nam, Dexter, and Sapirstein (2005), with a minor modification: 2 g instead of 200 mg sample was used. Cut fresh Chinese mushrooms were ground in 20 ml of a precooled 1% HCl‐methanol solution and placed at 4°C in the dark for 1 hr for extraction. The extract was filtered, the supernatant was collected, and its absorbance at 280 nm was determined using 1% HCl‐methanol solution as a baseline standard.

PPO activity was determined using the method of Mishra, Gautam, and Sharma (2013) with a slight modification: 5.0 g instead of 30 g of sample was used. Mushroom sample was added to 50 mmol L^−1^ phosphate buffer solution (containing 1% PVP, pH 5.5), ground on ice, and centrifuged at 4°C at 15,000 × *g* for 20 min. Supernatant (0.1 ml) of this enzyme solution was added to 3.9 ml of phosphate buffer (50 mmol L^−1^, pH 6.5), and 1 ml of 0.1 mol L^−1^ catechol solution was added. After 10 min at 37°C, 20% TCA solution was added to terminate the reaction. The mixture was centrifuged at 5000 × *g* at 4°C for 10 min. Enzyme activity was detected by determining the absorbance of the solution at 420 nm. An enzyme solution that had been boiled for 5 min was used as a negative control. One unit of enzymatic activity was defined as the amount of enzyme which caused a change in absorbance of 0.01/min. The PPO activity was expressed as U per gram of mushroom weight. The PPO specific activity was expressed as PPO activity g^−1^ protein (U g^−1^ protein).

### 2.8 Statistical analysis

Data were subjected to analysis of variance (ANOVA), and the means were compared by multiple‐range least significant difference (LSD) tests using DPS V3.01. *p *<* *0.05 was considered significant, and *p *<* *0.01 was considered extremely significant. Correlations between browning degree and Vc content, total phenol content, and PPO activity were analyzed in DPS V3.01.

## 3 RESULTS

### 3.1 Sensory quality

After 3 days of storage, the control mushrooms appeared to shrink, showed browning at the bottom, and had an unpleasant odor. After 4 days, control mushrooms completely lost edible value, with rotting odor and dark brown discoloration. On the other hand, mushrooms pretreated with paper containing 1‐MCP showed no significant difference when compared to fresh Chinese mushrooms. The sensory evaluation results of mushrooms at 4 days after treatments are listed in Table 1. After 1‐MCP treatment, Chinese mushrooms maintained better appearance, color, texture, and flavor. Best scores for appearance, firmness, and flavor were obtained with paper containing 0.5 and 0.75 μl L^−1^ 1‐MCP and best scores for color representing the color closest to the fresh mushroom with paper containing 0.5, 0.75, and 1 μl L^−1^ 1‐MCP. Taken together, paper containing 1‐MCP at a concentration of 0.75 μl L^−1^ was the most effective at maintaining quality and flavor, with no significant difference compared to fresh mushrooms.

**Table 1:** Sensory scores for Chinese mushroom fruiting bodies stored at 15 ± 1°C for 4 days

Treatment concentrations μl L^−1^	Sensory evaluation index			
	Appearance	Color	Firmness	Flavor
Control	1.2 ± 1.4^d^	0.8 ± 0.2^d^	5.3 ± 1.5^c^	2.2 ± 1.3^c^
0.25	7.6 ± 2.4^b^	7.1 ± 0.9^b^	8.4 ± 1.0^b^	6.8 ± 0.9^b^
0.50	8.3 ± 4.2^b^	8.8 ± 0.9^a^	9.2 ± 0.8^a^	8.3 ± 1.0^a^
0.75	9.8 ± 0.2^a^	9.3 ± 0.7^a^	9.4 ± 0.2^a^	8.6 ± 0.7^a^
1.00	9.8 ± 0.2^a^	8.6 ± 0.4^a^	8.5 ± 0.7^b^	7.2 ± 0.3^b^
1.25	6.4 ± 1.8^c^	6.2 ± 0.8^c^	8.1 ± 2.0^b^	6.9 ± 1.4^b^

*Note*. Mean ± *SE* scores (*n *= 10). ^a–d^ Means ± *SE* followed by the same letter, within a column, are not significantly different (*p *> 0.05).

### 3.2 Weight loss and firmness

**Figure 1:**
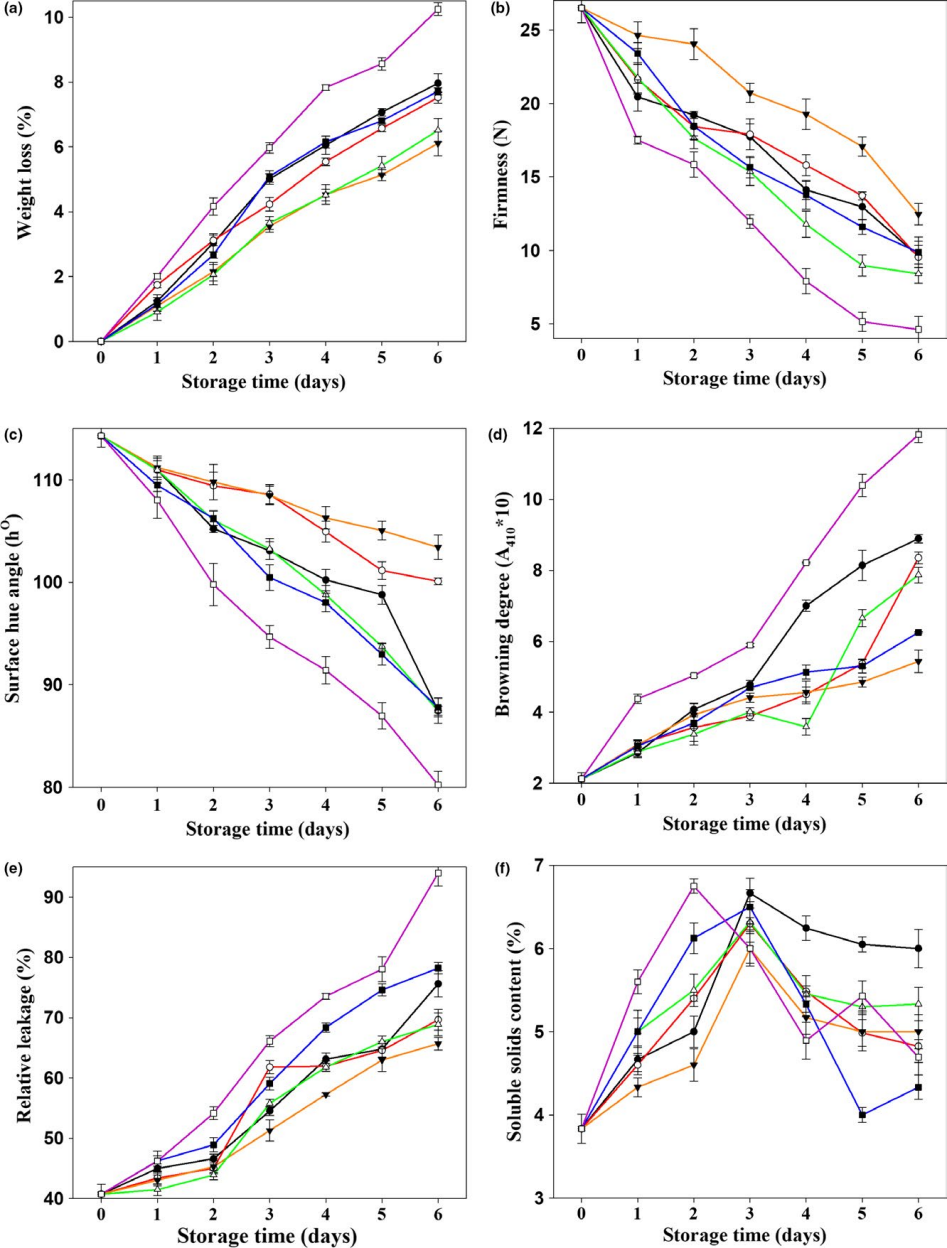
Effects of paper containing 1‐MCP at various concentrations on different quality features of Chinese mushroom fruit bodies. (a) Weight loss; (b) firmness; (c) surface hue angle (h°); (d) browning degree; (e) relative leakage rate; and (f) soluble solid content of Chinese mushrooms treated (□) 0, (●) 0.25, (○) 0.50, (▼) 0.75, (△) 1.00, and (■) 1.25 μl L^−1^ 1‐MCP. Vertical bars represent standard errors of the means (*n *= 5)

The weight loss was calculated daily throughout storage. The mushrooms not treated with 1‐MCP had deteriorated by day 4 and were inedible. To facilitate the comparison of experimental results, mushrooms were stored for 6 days. As shown in Figure 1a, the weight loss of mushrooms increased over storage time. Treatment with different concentrations of 1‐MCP effectively reduced the weight loss to a level lower than that of the control group, suggesting that 1‐MCP treatment can delay weight loss during storage. Statistical analysis showed that the weight loss of 1‐MCP‐treated mushrooms was extremely significant lower (*p *<* *0.01) than that of the control group over the entire storage period. These results indicated that 1‐MCP treatment effectively reduces the weight loss of Chinese mushrooms, thus extending their freshness. The most effective concentrations of 1‐MCP were between 0.75 μl L^−1^ and 1.00 μl L^−1^.

The firmness of 1‐MCP‐treated mushrooms was extremely significant higher (*p *<* *0.01) than that of control mushrooms, indicating that 1‐MCP treatment can maintain the firmness of Chinese mushrooms during storage. As shown in Figure 1b, the firmness declined with prolonged storage. Treatment with different concentrations of 1‐MCP resulted in differences in firmness. Throughout storage, the firmness of mushrooms in the 0.75 μl L^−1^ 1‐MCP treatment group was higher than that under the other four treatments.

### 3.3 Surface hue angle (h°)

The h° in 1‐MCP‐treated mushrooms was higher than those of the control group during storage. As shown in Figure 1c, the h° of the Chinese mushrooms decreased with the extension of storage time, with the most rapid decrease observed in the control group, which suggests that 1‐MCP treatment can slow down surface color change. The strongest effect was observed at 1‐MCP concentrations between 0.75 μl L^−1^ and 1.00 μl L^−1^.

As shown in Figure 1d, the browning degree of Chinese mushrooms increased in general; the 0.25 μl L^−1^ 1‐MCP treatment group exhibited significantly less (*p *<* *0.05) browning as compared to the control group. The other concentrations exerted even extremely significant stronger (*p *<* *0.01) browning‐suppressive effects during storage. On the sixth day, the browning degree after treatment with 1‐MCP was extremely significant lower (*p *<* *0.01) than that in the control group (11.82 ± 0.22) at all concentrations tested.

### 3.4 Cellular membrane permeability and respiration rate

As seen in Figure 1e, the leakage rate during the storage of Chinese mushrooms generally increased over time. Exposure to 1.25 μl L^−1^ 1‐MCP, as well as the other treatments, significantly slowed this process compared to the control (*p *<* *0.05 and *p *<* *0.01, respectively). On the sixth day, the leakage rate in all treatment groups was extremely significant lower (*p *<* *0.01) than that in the control group (94 ± 2%). Paper containing 1‐MCP at 0.75 μl L^−1^ had the strongest leakage rate‐maintaining effect. This indicates that 1‐MCP treatment could effectively retard the destruction of cellular membrane structure in the pulp of harvested mushrooms.

**Figure 2:**
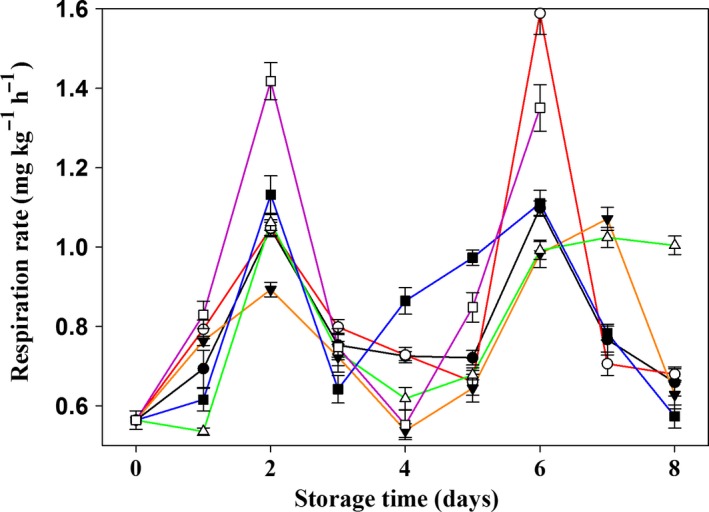
Effects of paper containing 1‐MCP at various concentrations on the respiration rate of Chinese mushrooms

To detect the respiration type of Chinese mushrooms, we assessed respiration rates until 2 days after the control samples had completely lost edible value. The respiration rate of CM in this study was in accordance with the rate typical for climacteric mushrooms. The first respiration climacteric peak appeared on the second day, and the second peak appeared on days 6–7. As shown in Figure 2, the intensities of these two peaks were similar. Compared with the control group, the first peak for 1‐MCP‐treated mushrooms was lower. On the second day, the respiration rate for the 0.75 μl L^−1^ and 1.00 μl L^−1^ 1‐MCP‐treated groups was 0.89 ± 0.02 mg kg^−1^ hr^−1^ and 1.06 ± 0.02 mg kg^−1^ h^−1^, respectively, which was lower than that for the control group (1.42 ± 0.05 mg kg^−1^ hr^−1^). These respiration rates were significantly decreased (*p *<* *0.05) by 58.87% and 33.52% as compared to that for the control. On day 6, the respiration rates of groups treated with 0.75 μl L^−1^ and 1.00 μl L^−1^ 1‐MCP were 0.98 ± 0.03 mg kg^−1^ hr^−1^ and 0.99 ± 0.02 mg kg^−1^ hr^−1^, respectively, indicating reductions of 27.21% and 26.58%, compared to the control group (1.35 ± 0.06 mg kg^−1^ hr^−1^). Thus, 1‐MCP treatment effectively reduced the respiration rate of Chinese mushrooms and had an anti‐aging effect. The most effective concentration of 1‐MCP in terms of these features was found to be between 0.75 μl L^−1^ and 1.00 μl L^−1^.

### 3.5 Soluble solid content

Soluble solids can be used to evaluate the maturity of mushrooms. As shown in Figure 1f, the soluble solid content rose and fell over time as expected, with the control group reaching a maximum value on day 2 and the 1‐MCP‐treated groups showing a peak on day 3. These results show that 1‐MCP treatment inhibits the increase in soluble solids and delays senescence to some extent in Chinese mushrooms at the first few days of storage. In both the treatment groups and the control group, the soluble solid content began to decline after it reached a peak. The soluble solid content was higher in the 0.25 μl L^−1^ 1‐MCP treatment group than in the control group, with the other treatment groups showing no obvious differences compared to the control group. There was a significant difference between the control group and the treatment groups in the first 2 days (*p *<* *0.05) while there was no significant difference over the total of 6 days (*p *>* *0.05), suggesting that early 1‐MCP treatment can inhibit the increase in the content of soluble solids and the senescence of CM, but late in storage, the effect is poor.

### 3.6 Vc, phenolics, and PPO activity

**Figure 3:**
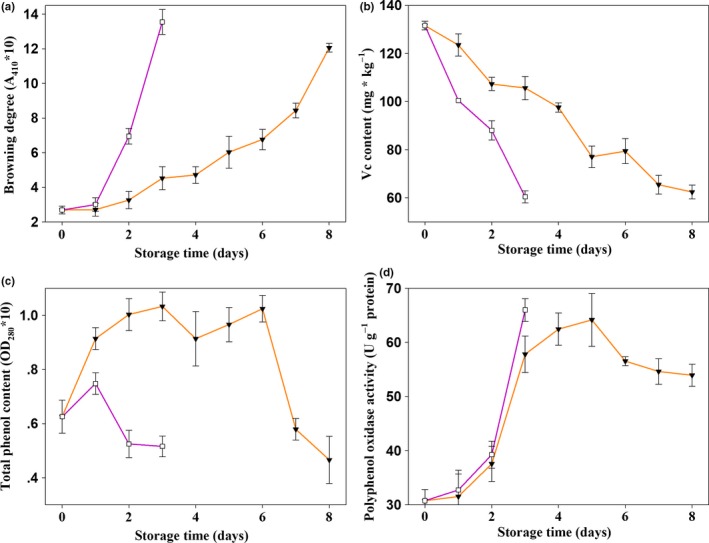
Effect of paper containing 1‐MCP at various concentrations on features related to browning of Chinese mushroom fruit bodies. (a) Browning degree; (b) vitamin C (Vc) concentration; (c) total phenol concentration; and (d) polyphenol oxidase activity of Chinese mushrooms treated with 0 (□) or 0.75 μl L^−1^ (▼) 1‐MCP. Vertical bars represent standard errors of the means

Browning was delayed by 1‐MCP (Figure 3a). As shown in Figure 3b, the Vc content of Chinese mushrooms decreased with the increase in storage time. During the entire storage period, the Vc content in the control group decreased rapidly, while the levels in the treatment group decreased relatively slowly. On day 3, the Vc content in the combined treatment groups and the control group was 106 ± 5 mg kg^−1^ and 60 ± 3 mg kg^−1^, respectively, which were lower than the levels on day 0 (132 ± 2 mg kg^−1^, decreases of 20% and 55%, respectively). Variance analysis showed that this difference was significant (*p *<* *0.05).

Total phenol contents tended to initially increase and then decrease (Figure 3c). The total phenol content in the control group increased after the first day and then declined, while that in 1‐MCP treatment increased between day 0 and day 6 (although it decreased slightly on day 4) and eventually decreased after day 6. Statistical analysis showed that there was a significant difference between the two groups (*p *<* *0.05).

PPO is an important enzyme involved in the browning process. As shown in Figure 3d, PPO activity was up‐regulated, with browning over the first 5 days in both the treatment and control groups. The PPO activity did not differ between the treatment and control groups during the first 2 days. However, variance analysis showed a significant difference between the treatment and control groups on the third day (*p *<* *0.05).

Correlation analysis indicated that the browning degree was negatively correlated with Vc content (*p *<* *0.01) and total phenol content (*p *<* *0.05), and extremely significant positively correlated with the change in PPO activity (*p *<* *0.01). These results suggested that browning in Chinese mushrooms is related to the Vc content, total phenol content, and PPO activity.

## 4 DISCUSSION

In this study, the effects of exposing CM to paper containing 0.25, 0.50, 0.75, 1.00, and 1.25 μl L^−1^ 1‐MCP on comprehensive quality indexes were evaluated to test the effectiveness of this compound in preserving CM. The results showed that 1‐MCP reduced weight loss, browning degree, and leakage rate. In addition, 1‐MCP treatment reduced the respiratory peak, mushroom body surface hue angle h°, and the soluble solid content, and maintained firmness as well as the original quality and flavor by inhibiting senescence and autolysis. Among the various concentrations, 0.75 μl L^−1^ 1‐MCP yielded the most promising results, extending the shelf life of CM from 2–3 days to 8 days, an extremely remarkable effect. Because paper containing 1‐MCP leaves no residue and storage in this paper is a low‐cost and simple operation (Chen et al., 2015), it can be expected to be widely applied to preserve CM if approval is obtained. These results provide a theoretical basis for the potential use of paper containing 1‐MCP in the development of CM commercial production.

How ethylene signaling plays a role in the senescence of mushrooms is unknown. Meng, Shen, Yang, Zhang, and Sheng (2014) identified the gene encoding 1‐aminocyclopropane‐1 carboxylate oxidase, a key enzyme for ethylene synthesis, in *A. bisporus*, which indicated that similar to plants, ethylene in mushrooms is synthesized via the methionine cycle. Our unpublished data showed that ethephon treatment could accelerate the ripening and senescence of Chinese mushrooms. Combined with the findings in this study, these results suggest that ethylene plays an important role in mushroom ripening. Similar with findings in various fruits and vegetables (Cai et al., 2006; Zhang, Tian, Zhu, Xu, & Qin, 2012), 1‐MCP treatment could delay postharvest browning of CM during storage by inhibiting PPO activity (Jamjumroon et al., 2013). However, the mechanism by which 1‐MCP could inhibit PPO activity remains unknown. In addition, browning in Chinese mushrooms was more strongly correlated with the effect of 1‐MCP treatment than with PPO activity, which further indicated that 1‐MCP treatment may suppress the ethylene signal transduction pathway in Chinese mushrooms, thus delaying maturation and senescence processes. In future studies, the genes involved in the ethylene signal transduction pathway in Chinese mushrooms should be identified and functionally analyzed to reveal the mechanisms underlying maturation and senescence.

## CONFLICT OF INTEREST

The authors declare that there is no conflict of interest.

## ETHICAL STATEMENT

This study does not involve any human or animal testing.

## AUTHOR CONTRIBUTIONS

Bingzhi Chen, Guohong Wu, and Ling Li were considered as co‐first author of this work. Bingzhi Chen and Yuji Jiang designed the study and interpreted the results. Qi Wei, Xiaoning Chen, Qiangui Zhong, Ling Li, and Kaiqian Xiao performed the experiments and collected test data. Bingzhi Chen, Yuji Jiang, and Baogui Xie drafted and revised the manuscript.
